# Protein Intrinsic Disorder and Evolvability of MERS-CoV

**DOI:** 10.3390/biom11040608

**Published:** 2021-04-20

**Authors:** Vladimir N. Uversky, Elrashdy M. Redwan, Abdullah A. Aljadawi

**Affiliations:** 1 Biological Science Department, Faculty of Science, King Abdulaziz University, P.O. Box 80203, Jeddah 21589, Saudi Arabia; 2 Department of Molecular Medicine and USF Health Byrd Alzheimer’s Research Institute, Morsani College of Medicine, University of South Florida, Tampa, FL 33612, USA

Middle East Respiratory Syndrome (MERS) is a viral respiratory disease caused by one of the human coronaviruses, MERS-CoV. Although MERS-CoV is similar to severe acute respiratory syndrome coronavirus (SARS-CoV), there is a noticeable difference between MERS-CoV and SARS-CoV and between MERS-CoV and the common-cold coronaviruses [1]. Most people infected with MERS-CoV develop a severe acute respiratory disease with fever, cough, and shortness of breath. This pathology rapidly progresses to pneumonia, and about half of the infected people die (the global mortality rate among patients is around 35%). Severe complications, including renal failure and acute respiratory distress syndrome (ARDS), have been commonly reported, with the ARDS being the main cause of the MERS-related mortality, which actually represents a common immunopathological event for SARS-CoV, MERS-CoV, and SARS-CoV-2 infections. Although MERS-CoV was first identified in Saudi Arabia in 2012, and although the outbreaks of MERS-CoV-related disease have been recorded in Saudi Arabia and the Republic of Korea, it is not clear as of now if MERS-CoV cases are slowing down or speeding up. All reported cases have had a direct or indirect link to one of four countries in the Middle East (Saudi Arabia, Qatar, United Arab Emirates, and Jordan), with reported patients either having lived in or travelled to these countries or having had close contact with people who acquired the infection there. Most reported MERS cases (above 80% of laboratory-confirmed cases) are from Saudi Arabia, occurring as sporadic, family, or hospital clusters with an approximate mortality rate of 50% [2]. There are no reported cases in Australia and the United States. Although MERS-CoV can be transmitted from human to human [3,4,5], the efficiency of MERS-CoV transmissibility is noticeably lower than that of SARS-CoV, which caused an epidemic of severe lower respiratory tract infections in 2002–2003 with a mortality rate of about 10% [6,7]. 

No efficient treatment or prevention of the MERS-CoV infection is available as of yet, and there are no pathogen-specific interventions that can be used for this disease. Consequently, patient management mainly depends on providing general support and caution for and prevention of complications. In specific situations, additional interventions have been included, such as the use of broad-spectrum anti-microbial agents, anti-virals, and anti-fungal agents to minimize the risk of co-infections with opportunistic pathogens. A variety of therapeutic agents have been screened against MERS-CoV, and several agents have shown inhibitory effects against MERS-CoV in vitro.

Studies showed that MERS-CoV is a zoonotic virus, and its presumed origin is in bats [8,9,10]. Evidence points to the occurrence of MERS-CoV infection in camels. In fact, specific antibodies against MERS-CoV spike protein were detected in 50 of 50 (100%) blood sera from retired racing Omani camels and in 15 of 105 (14%) samples from the Spanish camels. Furthermore, more than 90% of dromedary camels from Egypt were found to have antibodies reactive with MERS-CoV using various methods, including neutralization [11,12]. No such antibodies were found in blood sera from European goats, cattle, sheep, or other camelids [11]. Camels are economically important for people in the region, providing milk, meat, and recreation. Both Saudi Arabia and the United Arab Emirates produce and consume large amounts of camel meat, and it is possible that African or Australian bats harbor the virus, then camels carry it to the Middle East. In October 2013, a study provided virological confirmation of the presence of MERS-CoV in dromedary camels from a farm in Qatar linked to two human cases of the infection and suggested a recent outbreak affecting both humans and camels. However, currently, there is no definitive proof that camels are the source of infection for human cases of MERS. One cannot confirm whether the people on the farm were infected by the camels or vice versa, or if a third source was responsible [13]. 

MERS-CoV is an enveloped human coronavirus with a positive single-stranded RNA genome with a length of about 30 kb. The genome contains at least 11 open-reading frames (ORFs) that are translated into non-structural proteins (ORF1AB, ORF1A, NS3, NS4A, NS4B, NS5, and ORF8B) and structural proteins (spike (S), envelope (E), membrane (M), and nucleocapsid (N)) [14]. Structural proteins are the major constituents of the virion particle and encapsulate the viral genome. Characteristic spikes on the virus body (viral corona) are formed by the S protein, which is a transmembrane glycoprotein expressed on the surface of the virus envelope that has been studied as a candidate target for vaccine development [15]. Among the important roles of this protein are virus entry, receptor binding, and membrane fusion [14]. Although virus assembly, budding, and intracellular trafficking are dependent on the envelope small membrane protein (E), which is highly expressed in the infected cell, the exact role of this membrane protein during infection is not completely understood [14]. Being the most abundant protein component of the MERS-CoV envelope, the membrane protein (M) is mostly responsible for viral assembly and envelope formation [16]. The nucleocapsid protein (N) is a viral RNA-binding protein that forms a ribonucleoprotein (RNP) complex with the RNA genome and plays important roles in viral assembly and replication [14]. The major functions of the viral non-structural proteins, which are expressed in the infected cells, are related to the regulation of the replication and assembly of the virus [14].

Previous studies highlighted the importance of the intrinsic disorder for the functionality of MERS-CoV proteins [17], and peculiarities of the intrinsic disorder distribution within the inner and outer shell proteins were attributed to the high oral–fecal transmission potential of MERS-CoV [18]. However, despite the fact that all MERS-CoV proteins were shown to contain functionally important intrinsically disordered regions, no information is available on the potential role of intrinsic disorder in the evolvability of this virus. To address this issue, we generated the intrinsic disorder profiles for MERS-CoV and related proteins isolated from different infected hosts, such as bats (*Vespertilio sinensis*, *Pipistrellus kuhlii*, and *Neoromicia capensis* strains Neoromicia/5038 and Neoromicia/PML-PHE1/RSA/2011), camels (*Camelus dromedarius*), and humans (*Homo sapiens*), and also looked at changes in the intrinsic disorder predisposition of viral proteins from different MERS-CoV isolates. The corresponding protein sequences were retrieved from the MERS coronavirus database at the NCBI Virus Variation Resource (https://www.ncbi.nlm.nih.gov/genome/viruses/variation/) (accessed on 19 March 2021) [19]. 

Figure 1 represents the intrinsic disorder profiles for 11 viral proteins isolated from different MERS-CoV or MERS-CoV-related beta-coronaviruses infecting different hosts. It can be seen that irrespective of their origin, all proteins of the same type are characterized by remarkably similar intrinsic disorder profiles, emphasizing their close evolutionary relatedness. However, from the perspective of their intrinsic disorder predisposition, all proteins from the MERS-CoV infecting humans are noticeably closer to the profiles of corresponding proteins from the MERS-CoV infecting camels than to the profiles of proteins from the analogous coronaviruses infecting bats. This interesting observation suggests a close evolutionary connection between MERS-CoV infecting humans and camels. 

Obviously, despite being very peculiar, these findings do not necessarily indicate that camels served as intermediate hosts in the transition of the zoonotic MERS-CoV from bats to humans. On the other hand, since camels might serve as a reservoir for MERS-CoV, they are expected to contain neutralizing antibodies against MERS-CoV, whereas other animals had no such antibodies. In addition to the conventional antibodies that are composed of two heavy and two light chains, camels produce single-domain antibodies (sdAbs) or nanobodies, which do not have light chains [20]. The variable region of heavy chains (VHH fragments) of such heavy-chain antibodies (HCAbs) is solely responsible for the antigen binding. sdAbs have unique structural features that make them attractive vehicles for numerous research and medical applications, such as low molecular weight, superior stability, good water solubility [21], low immunogenicity [22], and an amazing ability to bind antigens inaccessible to conventional antibodies. These sdAbs are able to penetrate into tissues and organs that human immunoglobulins were unable to, where they are also capable of completely neutralizing bacteria or virus activity [23,24]. All this defines a broad interest in the medical sector regarding these camel nanobodies [23,24]. 

Figure 1 also shows that the intrinsic disorder profiles of proteins from MERS-CoV infecting humans, camels, and bats preserve many specific features, suggesting that such evolutionary conserved features can be of functional importance. Figure 2 represents another angle in the relation between the intrinsic disorder of viral proteins and the evolvability of MERS-CoV within humans. Here, Figure 2A shows the distribution of the percent of predicted intrinsically disordered residues values, i.e., residues with intrinsic disorder scores exceeding a threshold of 0.5 (PPIDR) for 11 viral proteins from all the currently available isolates of MERS-CoV infecting humans. MERS-CoV proteins are characterized by rather different levels of intrinsic disorder, and these levels can noticeably change in proteins from different MERS-CoV isolates, reflecting the viral evolution, which is a consequence of mutations that arise within and spread between infected hosts. Figure 2B provides another view of this phenomenon showing the mean disorder score (MDS) vs. PPIDR dependence for all these proteins. Based on data shown in Figure 2 and further summarized in Table 1, one can conclude that different MERS-CoV proteins are differently affected by the evolution of this virus within humans. In fact, some proteins (such as ORF1A, S, E, and M) change very little, whereas the intrinsic disorder predispositions of other proteins undergo remarkable evolutionary changes. 

Curiously, there is no uniform direction in the changes of the disorder predisposition of 11 MERS-CoV proteins. This is illustrated by Table 1, where for each MERS-CoV protein, the PPIDR values of the most and the least disordered members of the set are compared with the PPIDR of the corresponding protein from the earliest MERS-CoV isolate (2012/06/19, shown in Table 1 as Italicized text). These data indicate that MERS-CoV fitness within the host can be tuned by mutations leading to both an increase and decrease in intrinsic disorder predisposition of viral proteins.

It is tempting to hypothesize that the time-dependent changes in the intrinsic disorder status of the individual MERS-CoV proteins reported here, as evidenced by the variation of the intrinsic disorder predisposition observed for individual proteins from different MERS-CoV strains, are not only an indication of the potential role of intrinsic disorder in the evolvability of this virus but could serve as a general illustration of the relevance of intrinsic disorder to the evolution of viruses to adapt to the host. Obviously, finding more exact correlations represents an important subject for subsequent studies.

## Figures and Tables

**Figure 1:**
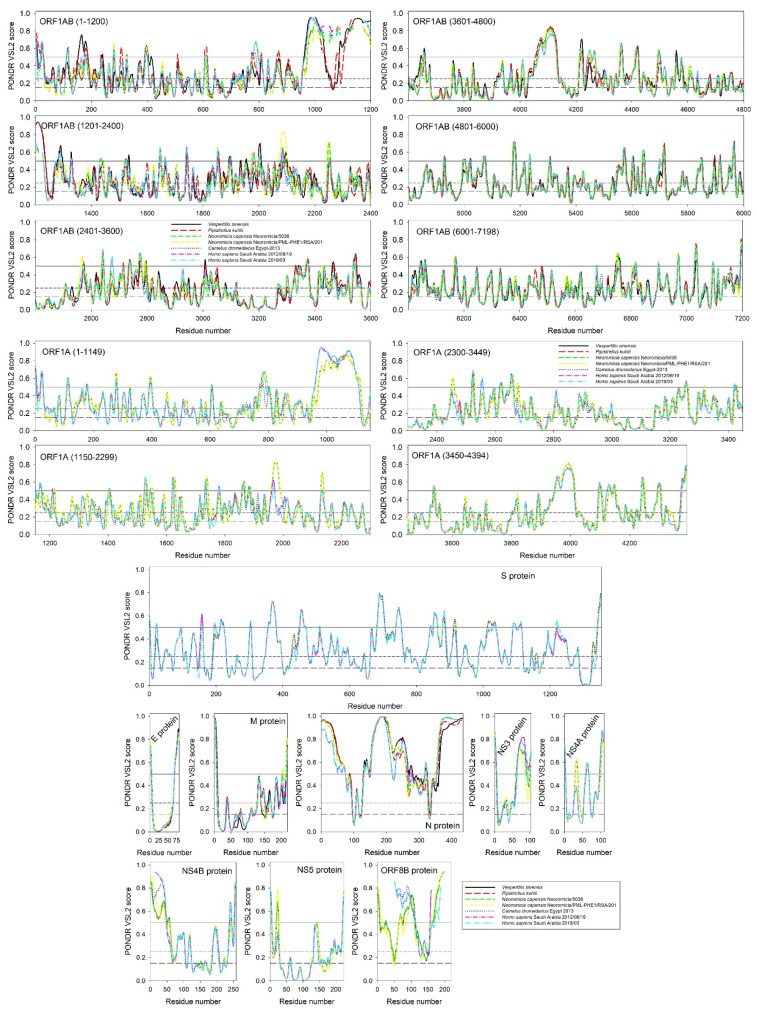
Intrinsic disorder profiles of 11 viral proteins from MERS-CoV infecting different species. Names of proteins are given inside the corresponding plots. Since ORF1AB and ORF1A are very long, their profiles are split into several windows. Note that no sequence information is available for some proteins from MERS-CoV infecting bats. For each of the 11 proteins from MERS-CoV infecting humans, we present two disorder profiles, showing disorder predisposition of the corresponding proteins found in the earliest and the latest isolates from Saudi Arabia (19/06/2012 and 03/2019, respectively).

**Figure 2:**
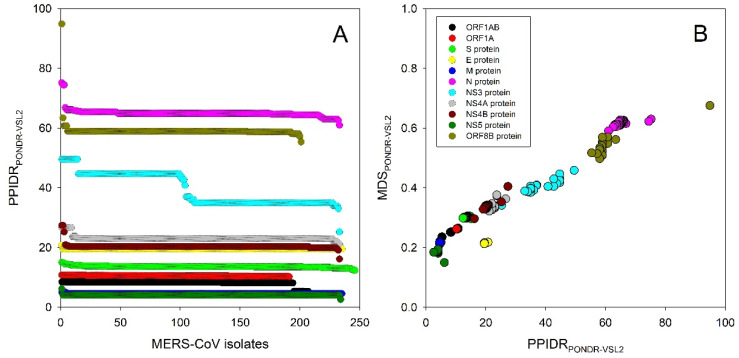
Evaluation of the effect of the viral evolution within humans on the intrinsic disorder predisposition of the MERS-CoV proteins. (**A**) Overall changes in the percent of predicted intrinsically disordered residues (PPIDR) values of 11 viral proteins from different MERS-CoV isolates. (**B**) MDS vs. PPIDR dependence for all these proteins. In this study, disorder predisposition was evaluated by the PONDR^®^ VSL2 predictor [25].

**Table 1:** Changes in the intrinsic disorder status of 11 proteins from various MERS-CoV isolates.

Protein	ID	PPIDR	Time	Protein	ID	PPIDR	Time
ORF1AB	*AGV08406*	*8.42%*	*2012/06/19*	**NS3**	*AGV08409*	*44.66%*	*2012/06/19*
	AIZ74438	5.30%	2013/05/07		AHE78109	33.01%	2013/11/05
	AFS88944	8.48%	2012/06/13		AGN70930	49.52%	2013/05/01
ORF1A	*AGV08407*	*10.66%*	*2012/06/19*	**NS4A**	*AGV08410*	*22.94%*	*2012/06/19*
	AIL23988	10.25%	2014/04/22		AMQ49006	21.10%	2014/11/04
	AWM99581	10.79%	2016/08/01		AKN24778	26.61%	2014/05/12
S protein	*AGV08408*	*13.52%*	*2012/06/19*	**NS4B**	*AGV08411*	*27.39%*	*2012/06/19*
	AHC74088	12.34%	2013/10/13		AIZ74435	16.15%	2013/05/07
	AMQ49004	14.98%	2014/11/04		AGV08382	27.39%	2012/10/23
E protein	*AGV08413*	*19.51%*	*2012/06/19*	**NS5**	*AGV08412*	*4.02%*	*2012/06/19*
	AIL23939	19.51%	2014/04/07		AIZ74454	2.56%	2013/05/07
	AGV08472	20.73%	2013/05/13		AMQ49019	6.12%	2015/07/12
M protein	*AGV08414*	*4.57%*	*2012/06/19*	**ORF8B**	*AGV08416*	*58.93%*	*2012/06/19*
	AKK52618	4.57%	2015/03/01		AXN92235	53.36%	2018/05/05
	ARQ84741	5.02%	2015/08/25		AIL23997	94.87%	2014/04/22
N protein	*AGV08415*	*64.89%*	*2012/06/19*				
	AIZ74430	60.99%	2013/05/07				
	AHI48709	75.18%	2013/08/06				

## Data Availability

Not applicable.
